# Mapping of a Chromosome 12 Region Associated with Airway Hyperresponsiveness in a Recombinant Congenic Mouse Strain and Selection of Potential Candidate Genes by Expression and Sequence Variation Analyses

**DOI:** 10.1371/journal.pone.0104234

**Published:** 2014-08-11

**Authors:** Cynthia Kanagaratham, Rafael Marino, Pierre Camateros, John Ren, Daniel Houle, Robert Sladek, Silvia M. Vidal, Danuta Radzioch

**Affiliations:** 1 Department of Human Genetics, McGill University, Montreal, Quebec, Canada; 2 Faculty of Medicine, Division of Experimental Medicine, McGill University, Montreal, Quebec, Canada; 3 Department of Microbiology and Immunology, McGill University, Montreal, Quebec, Canada; 4 Research Institute of the McGill University Health Center, Montreal, Quebec, Canada; 5 McGill University and Genome Quebec Innovation Centre, Montreal, Quebec, Canada; Tufts School of Medicine, United States of America

## Abstract

In a previous study we determined that BcA86 mice, a strain belonging to a panel of AcB/BcA recombinant congenic strains, have an airway responsiveness phenotype resembling mice from the airway hyperresponsive A/J strain. The majority of the BcA86 genome is however from the hyporesponsive C57BL/6J strain. The aim of this study was to identify candidate regions and genes associated with airway hyperresponsiveness (AHR) by quantitative trait locus (QTL) analysis using the BcA86 strain. Airway responsiveness of 205 F2 mice generated from backcrossing BcA86 strain to C57BL/6J strain was measured and used for QTL analysis to identify genomic regions in linkage with AHR. Consomic mice for the QTL containing chromosomes were phenotyped to study the contribution of each chromosome to lung responsiveness. Candidate genes within the QTL were selected based on expression differences in mRNA from whole lungs, and the presence of coding non-synonymous mutations that were predicted to have a functional effect by amino acid substitution prediction tools. One QTL for AHR was identified on Chromosome 12 with its 95% confidence interval ranging from 54.6 to 82.6 Mbp and a maximum LOD score of 5.11 (*p* = 3.68×10^−3^). We confirmed that the genotype of mouse Chromosome 12 is an important determinant of lung responsiveness using a Chromosome 12 substitution strain. Mice with an A/J Chromosome 12 on a C57BL/6J background have an AHR phenotype similar to hyperresponsive strains A/J and BcA86. Within the QTL, genes with deleterious coding variants, such as *Foxa1*, and genes with expression differences, such as *Mettl21d* and *Snapc1,* were selected as possible candidates for the AHR phenotype. Overall, through QTL analysis of a recombinant congenic strain, microarray analysis and coding variant analysis we identified Chromosome 12 and three potential candidate genes to be in linkage with airway responsiveness.

## Introduction

The assessment of airway responsiveness to methacholine is one of the key tests for diagnosing asthma. Airways naturally respond to stimuli such as methacholine by constricting, resulting in decreased airflow to the lungs. In asthmatic patients, this response occurs more quickly and forcefully, and at lower doses of the airway constricting agent. This heightened response is known as airway hyperresponsiveness (AHR).

Studies involving murine models have shown that mice have an airway responsiveness phenotype comparable to humans [1]. Certain inbred strains, such as the A/J strain, have an airway hyperresponsive phenotype when exposed to methacholine and can be used as a model to study the phenotype observed in asthmatic individuals. Other strains, such as the C57BL/6J and C3H/HeJ strains, are relatively less responsive to methacholine [2].

In addition to being able to model the airway responsiveness phenotypes seen in humans, mouse studies have also shown that airway responsiveness is a polygenic trait [1], [3]. Using crosses generated from inbred strains of mice, several quantitative trait loci (QTLs) for AHR in naïve, non-allergic mice have been identified. In crosses derived from A/J and C57BL/6J strains, QTLs for AHR were located on Chromosomes 2, 6, 15, and 17 [3], [4]. Interacting loci have also been identified on Chromosomes 2 and 6, and 11 and 18 [4], [5]. Similarly, using progeny from crosses of A/J and C3H/HeJ mice, genomic regions associated with AHR were found on Chromosomes 6 and 7, along with a suggestive association on Chromosome 17 [6], [7]. Mouse Chromosome 13 has been linked to AHR through recombinant inbred strains generated from C57BL/6 and DBA/2, and from haplotype association mapping using 36 inbred strains [2], [8].

This paper reports QTL mapping results using a unique inbred strain generated as part of a panel of AcB/BcA recombinant congenic strains (RCS). The RCS were created by backcrossing (A/JxC57BL/6J)F1 progeny to parental strains twice, followed by at least 30 generations of inbreeding. This resulted in 33 RCS each fully inbred and composed of approximately 12.5% of genetic material from one parental strain (minor genetic donor) and approximately 87.5% from the other (major genetic donor) [9]. The strength of the RCS panel as a genetic tool can be illustrated by the QTLs identified for various traits, such as susceptibility to infections by influenza, malaria, and salmonella, and in psychiatric studies like alcohol preference and response to stress [10]–[14].

The AHR trait segregates among the parental strains, A/J and C57BL/6J, of the AcB/BcA RCS. In a previous study, the entire AcB/BcA RCS panel had been phenotyped for airway responsiveness to methacholine [15]. Association analysis between the phenotypes and genotypes from the 33 RCS identified 16 chromosomal regions linked with AHR, of which eight were novel. In addition, certain strains were particularly informative because their phenotypes were significantly different from the phenotype of their major genetic donor strain, and were comparable to the phenotype of their minor genetic donor strain. Of the 22 BcA strains that were phenotyped, BcA86 was the most significantly different from its major genetic donor strain, C57BL/6J, and resembled the A/J strain phenotype [15]. We hypothesized that QTL analysis for AHR can be done using F2 progeny of the BcA86 strain (BcA86F2) to identify candidate regions and genes associated with AHR. The results presented in this paper describe in detail our strategy that allowed us to identify, a QTL on Chromosome 12 that is linked to AHR.

## Materials and Methods

### Animals

All experiments involving animals were approved by the McGill Animal Care Committee and were in compliance with the regulations set by the Canadian Council for Animal Care. All animals were housed in a biosecurity level 4 facility. List of excluded pathogens can be found at https://www.mcgill.ca/research/sites/mcgill.ca.research/files/611-_excluded_pathogens_-_rodent_facilities__2013.pdf.

### A/J, C57BL/6J, Chromosome 12 substitution strain, and BcA86 strains

Breeding pairs for A/J, C57BL/6J, and Chromosome 12 substitution strain (CSS12, C57BL/6J-Chr12^A/J^/NaJ) were purchased from The Jackson Laboratory (Bar Harbor, Maine, USA). The BcA86 strain used in this study is part of the AcB/BcA recombinant congenic strain panel generated at McGill University [9]. Colonies were housed (1–4 animals/cage), bred, and maintained at the Montreal General Hospital Research Institute in a pathogen free facility with a 12-hour light/dark cycle. All animals used in experiments were between eight and twelve weeks of age.

### Generation of BcA86F2 mice

BcA86 mice were backcrossed to C57BL/6J mice to generate F1 mice. The resulting F1 progeny were subsequently interbred (F1xF1) to create BcA86F2 mice. F1 progenies were generated with both male and female BcA86 and C57BL/6J mice.

### Measuring enhanced pause (Penh)

Penh, a dimensionless index of airway obstruction, was measured using an unrestrained whole body plethysmograph (Buxco Research System, Wilmington, NC, USA) in response to various doses of methacholine prepared in PBS. Mice were placed in the plethysmograph chamber and left to acclimate to the environment for five minutes before baseline Penh was measured. Mice were then exposed to increasing doses of methacholine (Sigma, St-Louis, Missouri, USA). 50ul of methacholine was aerosolized and nebulized into the plethysmograph chamber over one minute and the average Penh value during the exposure was used for analysis. Mice were only exposed to subsequent doses of methacholine once Penh values returned to baseline. Based on preliminary studies, the 15 mg/ml dose of methacholine was deemed the most reliable to compare Penh between A/J, C57BL/6J, BcA86 and BcA86F2 mice as it most consistently demonstrated the difference in phenotype between parental strains [15]. For experiments involving the CSS12 strain, a dose response curve, using 6.25, 12.5, 25, and 50 mg/ml of methacholine, was done to graphically visualize the segregation in airway responsiveness phenotype between C57BL/6J and CSS12.

### Measuring pulmonary resistance

Mice were anesthetized with a cocktail of ketamine (100 mg/kg), xylazine (10 mg/kg) and acepromazine (3 mg/kg). Once the depth of anesthesia was verified, the animals were tracheotomized and connected to a ventilator, and the baseline resistance values were measured. A nebulizer was used to administer PBS or a dose of 25 mg/ml methacholine directly to the lungs through the tracheostomy tube. The maximum resistance in response to the PBS or methacholine exposure for each mouse was determined using a Buxco plethysmograph system and Harvard Apparatus ventilators (Harvard Apparatus, Holliston, MA, USA).

### Tail DNA extraction and genotyping

Genotyping was done using tail DNA samples from BcA86F2 mice. Approximately 1mm of tail tissue was digested overnight in tail lysis buffer (5 M NaCl, 20% SDS, 1 M Tris HCl, 0.5 M EDTA) containing 0.5 mg/ml proteinase K (Life Technologies, Carlsbad, CA, USA). DNA was purified by serial phenol-chloroform extractions, precipitated in isopropanol, and resuspended in TE Buffer (10 mM Tris, 1 mM EDTA). DNA concentration was measured using Quant-iT DNA Assay Kit (Life Technologies). BcA86F2 mice were genotyped in recombinant regions of BcA86 with 41 microsatellites and 47 SNP markers, with an average spacing of 3.5 Mbp between markers (Table S1). Microsatellite and SNP genotyping were done at Laval University, and the McGill University and Genome Quebec Innovation Center, respectively.

### Microarray for selection of candidate genes with expression differences

Lungs were collected from euthanized animals and stored in RNA Later stabilization reagent (Qiagen, Venlo, Limburg, Netherlands). Approximately 30 mg of lung tissue was used for RNA extraction using the RNeasy Mini Kit (Qiagen) following the manufacturer's instructions. Samples which passed the quality control analysis on an Agilent Bioanalyzer (Santa Clara, CA, USA) were subsequently used with Affymetrix Gene 2.0 ST array (Affymetrix, Inc., Santa Clara, CA, USA). Three lung samples from each C57BL/6J, CSS12 and BcA86 strains were used. Quality control testing, cDNA preparation and hybridization were done at the McGill University and Genome Quebec Innovation Center.

All microarray datasets are available in the Gene Expression Omnibus database under accession number GSE52356. Raw data was normalized using the log2 expression values and robust multiarray average method (RMA) in FlexArray [16], [17]. Hyporesponsive and hyperresponsive strains were compared by Cyber T-test. Probesets were considered as significant if their log transformed fold changes were less than 0.71 or greater than 1.4 with *p* values <0.05. We selected protein coding genes as candidates if they were annotated as “validated” in the Reference Sequence database, and if they were differently expressed between C57BL/6J and CSS12, and C57BL/6J and BcA86 in the same direction, and not differently expressed between CSS12 and BcA86 [18]. Pathway analyses were done using Ingenuity Pathways Analysis (Ingenuity Systems, http://ingenuity.com). Network scores presented for significant pathways were calculated by the negative exponent of the right tailed Fisher's exact test.

### Quantitative RT-PCR for validation of microarray data

RNA transcript levels were measured using 1ug of total RNA, which was reverse transcribed into cDNA using the QuantiTect Reverse Transcription kit (Qiagen) according to the manufacturer's instructions. Equal amounts of cDNA were used to measure the relative expression of each candidate gene using a StepOne Plus Real Time QPCR instrument (Life Technologies) and Fast SYBR Green Master Mix (Life Technologies). Primers for each candidate gene were ordered from IDT DNA and the efficiency and specificity of the primers were verified by template dilution series analysis and melt curve analysis. Gene expression was normalized to the housekeeping gene *Gapdh* and relative expression was calculated using the cycle threshold (CT) values and the 2^-ΔΔCT^ method.

### Primer Sequences

*Eapp* forward: 5'-GGA TTG CAA AGG CCA CGT CAG AAA-3'

*Eapp* reverse: 5'-TGG CAA TCA AGG CAC AAT GTC GTC-3'

*Gapdh* forward: 5'-ATG TGT CCG TCG TGG ATC TGA-3'

*Gapdh* reverse: 5'-TTG AAG TCG CAG GAG ACA ACC T-3'

*Mettl21d* forward: 5′-ATGTGAACGGAGCATGTGCATACC-3′

*Mettl21d* reverse: 5′-TGTGGGCAGTCCAAAGTAGTTGTC-3′

*Snapc1* forward: 5′-AGT TGC CTT GAA GGA CTG GGA TGA-3′

*Snapc1* reverse: 5′-TAG CTG TGA AGT GGA AGG CTC TGT-3′

### *In silico* analysis of genotype-phenotype data and candidate gene selection using online databases

QTL analysis was done using the statistical software R, version 2.15.1. Association between marker genotype and phenotype was determined using the R/QTL library and by standard interval mapping to obtain LOD scores for each marker. To determine which markers were significant, 10,000 permutations of randomized genotype-phenotype combinations from our data were generated to obtain a LOD threshold. Markers were considered significant if their LOD scores were greater than the largest LOD score in 95% of the LOD values generated from the randomized data (*α* = 0.05). A 95% Bayes credible interval method was used to estimate the QTL location within the region harbored by the significant makers. Protein coding genes within the QTL containing coding non-synonymous SNPs (nsSNPs) between the A/J and C57BL/6J sequences were obtained using the Sanger dataset from the Mouse Phenome Database (http://phenome.jax.org/SNP). Polymorphisms were analyzed using PROVEAN and the HumDiv model of PolyPhen2 [19], [20].

### Statistical analysis

Unless otherwise specified, all other statistical analyses were done using GraphPad Prism 5.03 and *p*-values were obtained by one-way ANOVA followed by Bonferroni correction. Penh values were log transformed to provide a unimodal distribution of the phenotype. Normality of the Penh distribution was determined using the Kolmogorov–Smirnov test.

## Results

### Airway responsiveness of BcA86, BcA86F1, and BcA86F2 mice

**Figure 1:**
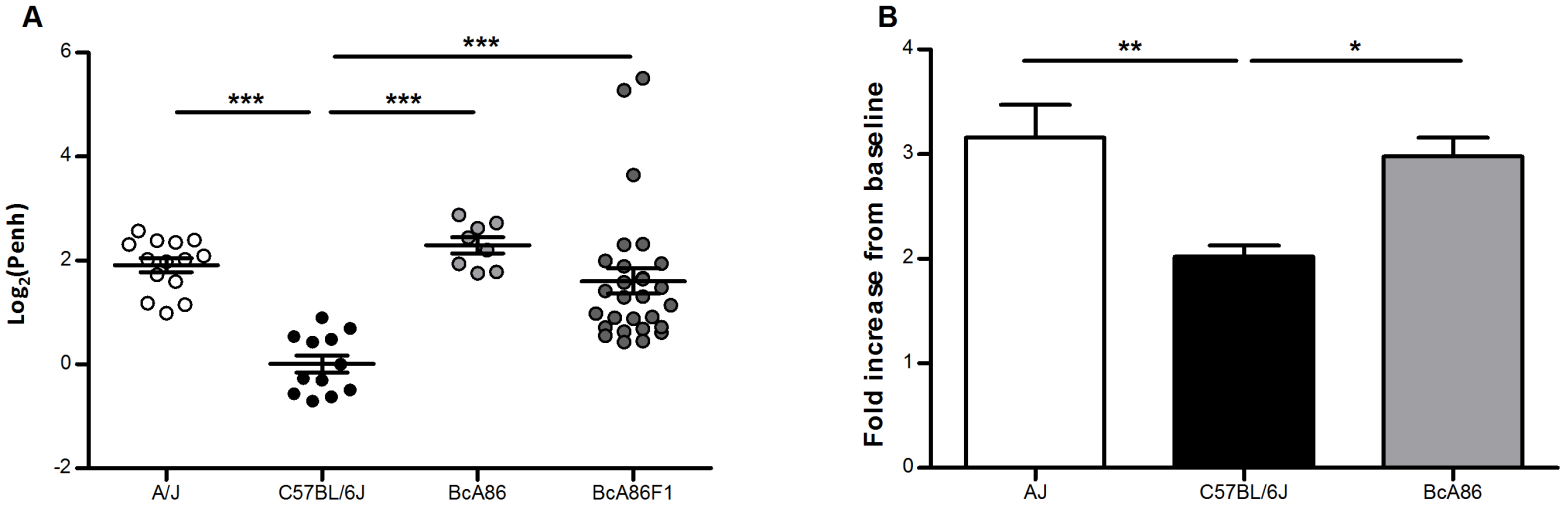
Airway responsiveness phenotype of BcA86 recombinant congenic strain . (A) Enhanced pause (Penh) in response to 15 mg/ml of methacholine was measured in A/J, C57BL/6J, BcA86 and BcA86 F1 mice. (n = 14 A/J, 12 C57BL/6J, 8 BcA86 and 28 BcA86F1). (B) Airway resistance of A/J, C57BL/6J and BcA86 strains in response to 25 mg/ml of methacholine. Resistance values were normalized by baseline readings and data is shown as the fold increase from baseline. Data are presented as mean ± SEM and statistical significance was calculated by one-way ANOVA and Bonferroni correction. n>5 per strain; _*, **_, and _***_ represent *p*<0.05, *p*<0.01, and *p*<0.001, respectively.

We determined the airway responsiveness of BcA86 strain relative to A/J and C57BL/6J strains by measuring Penh. We observed that BcA86 has an airway responsiveness phenotype similar to A/J and significantly different from C57BL/6J, confirming our previous results (Figure 1A) [15]. Similar observations can be made about BcA86F1 mice (Figure 1A).

**Figure 2:**
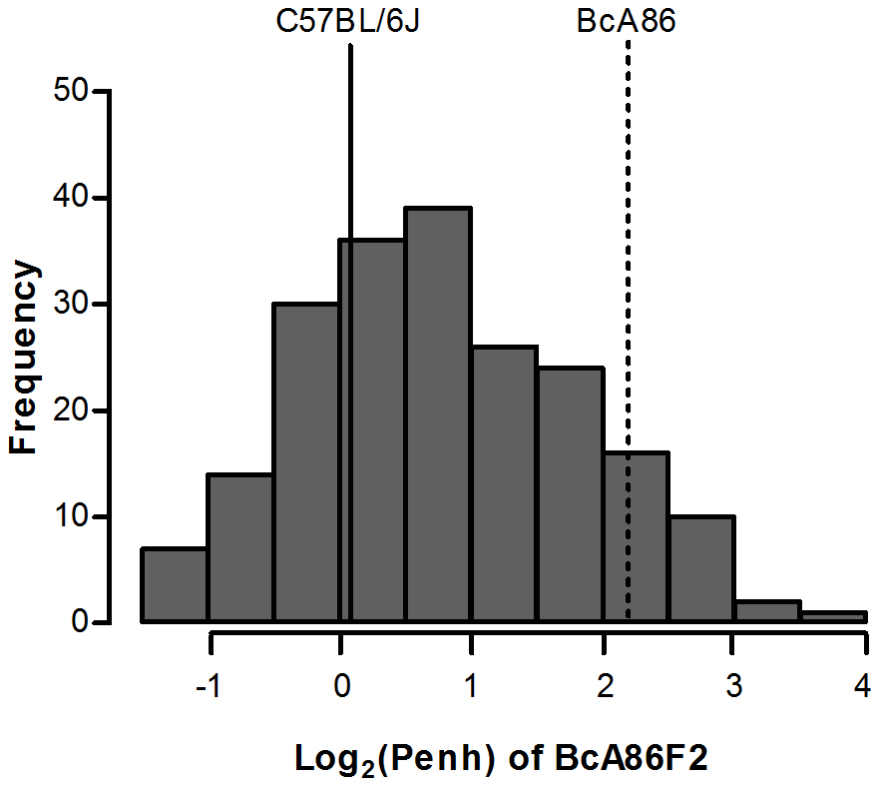
Histogram representation of the enhanced pause values of 205 BcA86F2. The distribution of log transformed enhanced pause values (log_2_(Penh)) of BcA86F2 mice ranges from lower than the average phenotype value of C57BL/6J (solid line) to greater than average phenotype value of BcA86 (dashed line).

Since Penh measurements are known to be influenced by factors other than airway resistance, we validated our observations from Penh with invasive direct measurements of airway resistance [21]. Our measurements confirmed that the BcA86 strain has a hyperresponsive phenotype similar to A/J strain (Figure 1B). To identify genetic determinants of airway responsiveness in the BcA86 strain, we generated 205 BcA86F2 mice for QTL mapping. The mean log_2_(Penh) of the BcA86F2 mice shows a transgressive pattern of segregation as their phenotypes range from lower than the mean log_2_(Penh) of C57BL/6J strain to higher than the mean log_2_(Penh) of the BcA86 strain (Figure 2). The normal distribution pattern of log_2_(Penh) values from the F2 mice supports the hypothesis that multiple interacting loci are involved in determining the airway responsiveness phenotype. We also observe that gain of C57BL/6J genomic material results in a greater airway responsiveness phenotype in some BcA86F2 mice in comparison to BcA86. This indicates that in addition to airway responsiveness protective loci, susceptibility loci are also found in the C57BL/6J genome.

### QTL mapping

**Figure 3:**
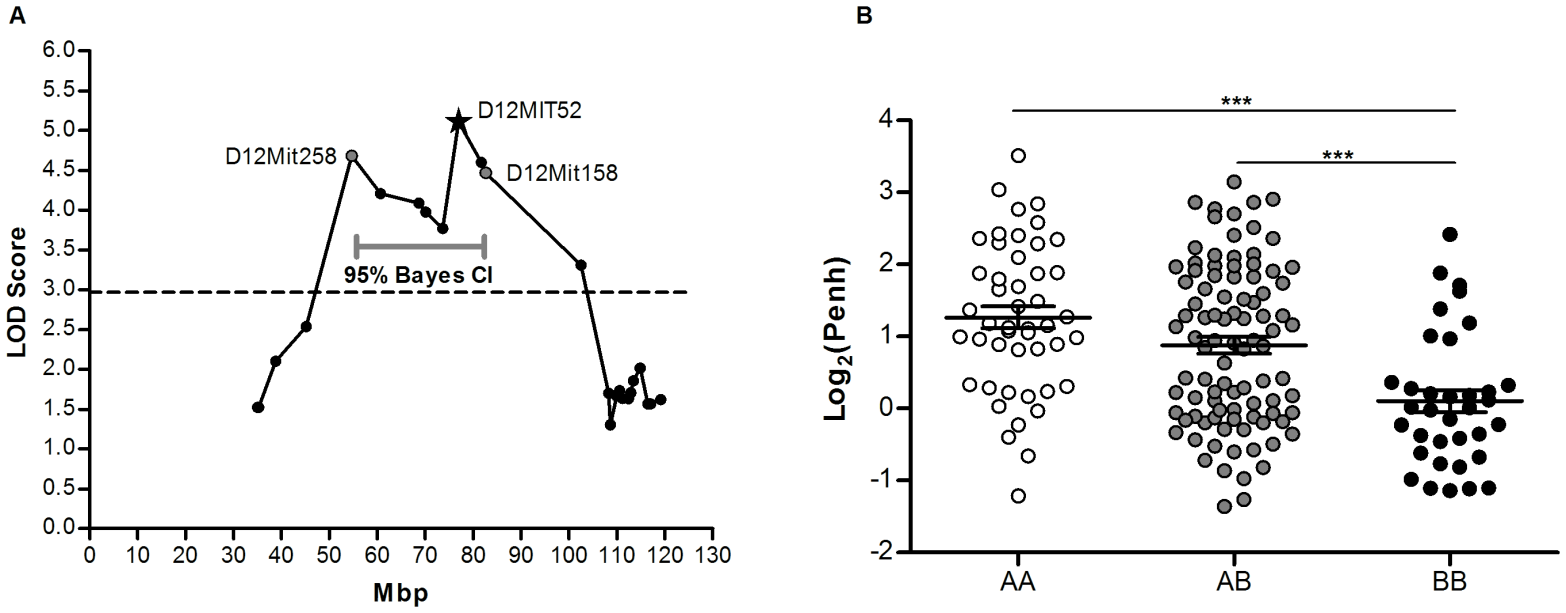
Association of Chromosome 12 markers with airway responsiveness in BcA86 mice. (A) Distribution of LOD scores of markers on Chromosome 12 used to genotype BcA86F2 mice. Dashed line at LOD  = 2.97 represents the 95% LOD threshold for significance generated from 10,000 permutations of randomized associations of the BcA86F2 genotype and phenotype data. Bayes 95% credible interval (CI) was used to determine the QTL confidence interval delimited by markers (grey dots) D12Mit285 at 54.6 Mbp (LOD score  = 4.68) and D12Mit158 at 82.6Mbp (LOD score  = 4.47). The most significant marker in the region is D12Mit52 (★) at 77.0Mbp with a LOD score of 5.11 and *p* = 3.68×10^−3^. (B) Distribution of log_2_(Penh) among the three possible genotypes at D12Mit52 (AA = homozygous for A/J, BB = homozygous for C57BL/6J and AB = heterozygous). Groups with A/J genotype at D12Mit52 (AA and AB) have significantly greater mean log2(Penh) value than homozygous C57BL/6J group (BB) (AA: 1.264 ± 0.151, AB: 0.879 ± 0.114, and BB: 0.102 ± 0.157). Data are presented as mean ± SEM and _***_ represents *p*<0.001 from one-way ANOVA and Bonferroni correction.

BcA86F2 mice were genotyped at 88 markers located within the previously identified nine recombinant regions of the BcA86 strain [10], [15]. To identify which genomic regions of BcA86 are associated with the AHR phenotype we did a QTL mapping analysis using the phenotype and genotype data of BcA86F2 mice. Of the 88 markers, nine consecutive markers on Chromosome 12 were significantly associated with the AHR phenotype, and surpassed the 95% threshold generated from 10,000 permutations (LOD threshold  = 2.97) (Table 1 and Table S1). One 28Mbp QTL was identified on Chromosome 12 whose 95% confidence interval was delimited on the left and right by markers D12Mit285 and D12Mit158, at 54.6Mbp and 82.6Mbp, respectively (Figure 3A). This QTL explains 12.5% of the phenotypic variance observed in the BcA86F2 mice. All eight markers within this region surpassed the 95% and 99% LOD threshold generated from 10,000 permutations (LOD threshold  = 2.97 and 3.65, respectively) (Table 1). The most significant marker among these eight markers is D12Mit52 at 77.0 Mbp, with a LOD score of 5.11 (*p* = 3.68×10^−3^). The distribution of phenotypes among the possible genotypes at D12Mit52 showed that the replacement of a C57BL/6J allele with an A/J allele causes an increase in log_2_(Penh). Mean log_2_(Penh) of animals with homozygous A/J genotype (AA) or heterozygous genotype (AB) was significantly higher than animals with homozygous C57BL/6J genotypes (BB) (Figure 3B). Two-QTL analysis did not identify any additional loci apart from the significant one on Chromosome 12.

**Table 1:** Significant markers from quantitative trait locus analysis.

Marker	Position (Mbp)	LOD score
**D12Mit285**	**54.6**	**4.68**
**D12Mit36**	**60.7**	**4.21**
**D12Mit71**	**68.6**	**4.09**
**D12Mit114**	**70.1**	**3.97**
**rs32323357**	**73.7**	**3.77**
**D12Mit52**	**77.0**	**5.11**
**rs29137109**	**81.7**	**4.60**
**D12Mit158**	**82.6**	**4.47**
D12Mit101	102.5	3.31

Significant markers have LOD scores greater than the 95% and 99% LOD thresholds generated from 10,000 permutations (LOD  = 2.97 and 3.65, respectively). Markers in bold are within the 95% confidence interval of the QTL.

### Mouse Chromosome 12 and airway responsiveness

**Figure 4:**
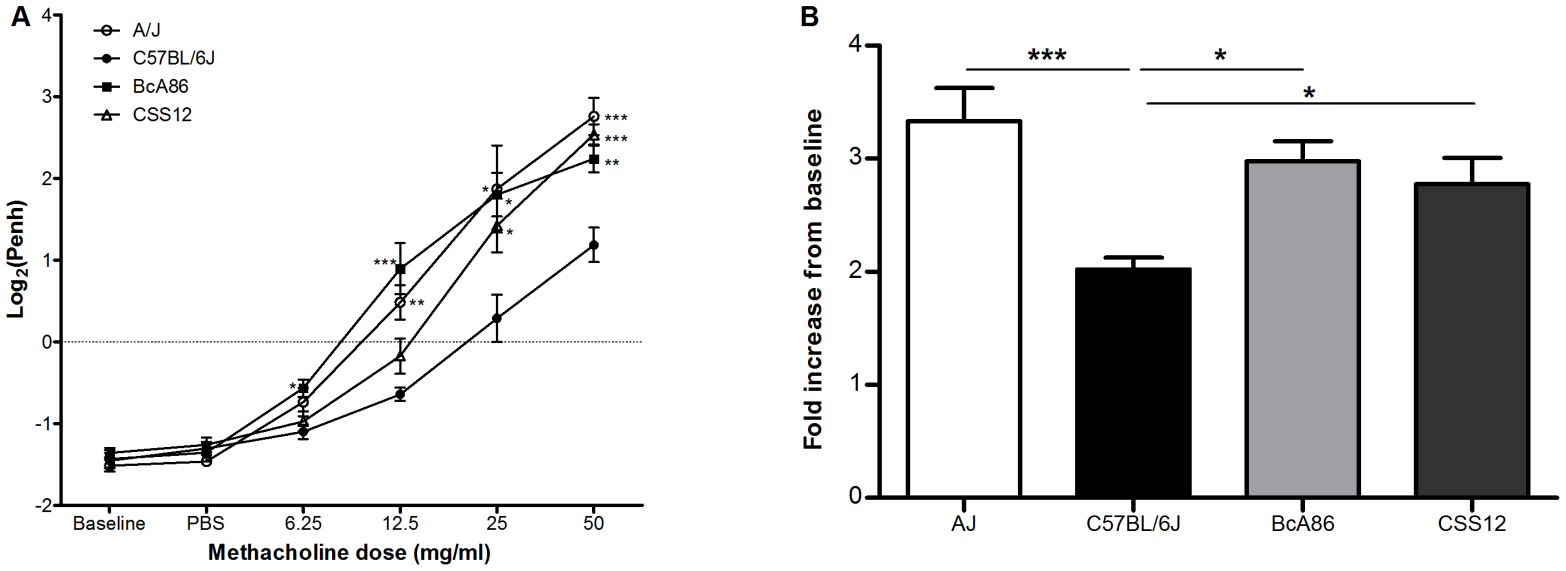
Genetic background of mouse Chromosome 12 determines airway responsiveness phenotype. (A) Log_2_(Penh) response curve to increasing doses of methacholine in A/J, C57BL/6J, BcA86, and CSS12 strains. The asterisks label the strains which are significantly different from C57BL/6J at each respective dose. (B) The airway resistance of A/J, C57BL/6J, BcA86 and CSS12 mice to 25 mg/ml of methacholine was measured by the gold standard invasive plethysmography and normalized to baseline readings. Data are presented as mean ± SEM and statistical significance was calculated by one-way ANOVA and Bonferroni correction. n>5 mice per strain for both experiments; _*, **_, and _***_ represent *p*<0.05, *p*<0.01 and *p*<0.001, respectively.

CSS12 mice have an A/J Chromosome 12 on a C57BL/6J background. We used CSS12 because it allowed us to see if an AHR phenotype can be observed in CSS12 in the absence of the other recombinant regions found in the BcA86 strain, or whether Chromosome 12 required the interaction with other loci found on other chromosomes for the phenotype to be expressed. CSS12 mice, along with A/J and BcA86 mice, were phenotyped for airway resistance by invasive and non-invasive methods and compared to hyporesponsive parental strain C57BL/6J. Penh values in response to increasing doses of methacholine show that the phenotypes of BcA86 and A/J segregate from C57BL/6J at doses as low as 6.25 and 12.5 mg/ml, respectively (Figure 4A). The phenotype of CSS12 is different from C57BL/6J starting from 25 mg/ml of methacholine. This difference in airway responsiveness between CSS12 and C57BL/6J and similarity between CSS12, BcA86, and A/J was validated by measuring respiratory system resistance to 25 mg/ml of methacholine using the invasive method (Figure 4B). Consistent with our previous findings obtained by measuring Penh, A/J, BcA86, and CSS12 strains had similar phenotypes (*p*  =  NS), and all three strains were significantly different from C57BL/6J.

### Pathway analysis of genes distinguishing hyporesponsive and hyperresponsive strains

We considered the possibility that regulatory polymorphisms may cause changes in expression of genes that could be involved in airway responsiveness. We performed a microarray experiment to compare the expression of genes in the lungs of a hyperresponsive strain in comparison to hyporesponsive strains. We chose to compare BcA86 and CSS12 to C57BL/6J, as these three strains shared a common genetic background. Genes thus identified are more likely to be associated to the phenotype being studied rather than differences in strain background and genetic heterogeneity. Hence, the candidate genes we selected had no significant difference in expression between the hyperresponsive strains BcA86 and CSS12. In addition, they had similar differences in expression (in value and direction), when comparing C57BL/6J to BcA86 and C57BL/6J to CSS12.

We identified 105 annotated genes that have similar expression levels between the hyperresponsive strains and different from the C57BL/6J strain (Table S2). Ingenuity pathway analysis revealed that the strongest networks representing these genes are “cell-mediated immune response” and “small molecule biochemistry” (Table 2). Diseases and functions analysis revealed that the genes are involved in the “proliferation and death of hematopoietic cells”, “development of muscle”, and “differentiation of connective tissue cells” (Table 3). “Airway pathology in COPD” was the top Ingenuity canonical pathway enriched by the 105 genes significantly different between hyperresponsive and hyporesponsive strains (*p* = 2.23×10^−2^).

**Table 2:** Pathway analysis: Top networks associated with genes distinguishing hyperresponsive strains from hyporesponsive strain.

Top Networks	Score
Cell-mediated Immune Response, Cellular Development, Cellular Function and Maintenance	30
Small Molecule Biochemistry, Molecular Transport, Hereditary Disorder	30
Cardiac Inflammation, Cardiovascular Disease, Inflammatory Disease	22
Cellular Movement, Immune Cell Trafficking, Hematological System Development and Function	15
Immunological Disease, Cellular Compromise, Cancer	8

Analysis was done using 105 genes (Table S2) distinguishing hyperresponsive strains, BcA86 and CSS12, from hyporesponsive strain, C57BL/6J.

**Table 3:** Pathway analysis: Top disease or functions distinguishing hyperresponsive strains from hyporesponsive strain.

Disease or Function	Category	*p*-value
Proliferation of hematopoietic cells	Cellular Development	1.91E-04
	Cellular Growth and Proliferation	1.91E-04
	Hematological System Development and Function	1.91E-04
	Hematopoiesis	1.91E-04
Cell death of hematopoietic cells	Cell Death and Survival	1.06E-03
Development of muscle	Embryonic Development	3.61E-04
	Organ Development	3.61E-04
	Organismal Development	3.61E-04
	Skeletal and Muscular System Development and Function	3.61E-04
	Tissue Development	3.61E-04
Differentiation of connective tissue cells	Cellular Development	1.30E-02

Analysis was done using 105 genes (Table S2) distinguishing hyperresponsive strains, BcA86 and CSS12, from hyporesponsive strain, C57BL/6J.

### Selection of candidate genes for airway responsiveness within QTL region

**Figure 5:**
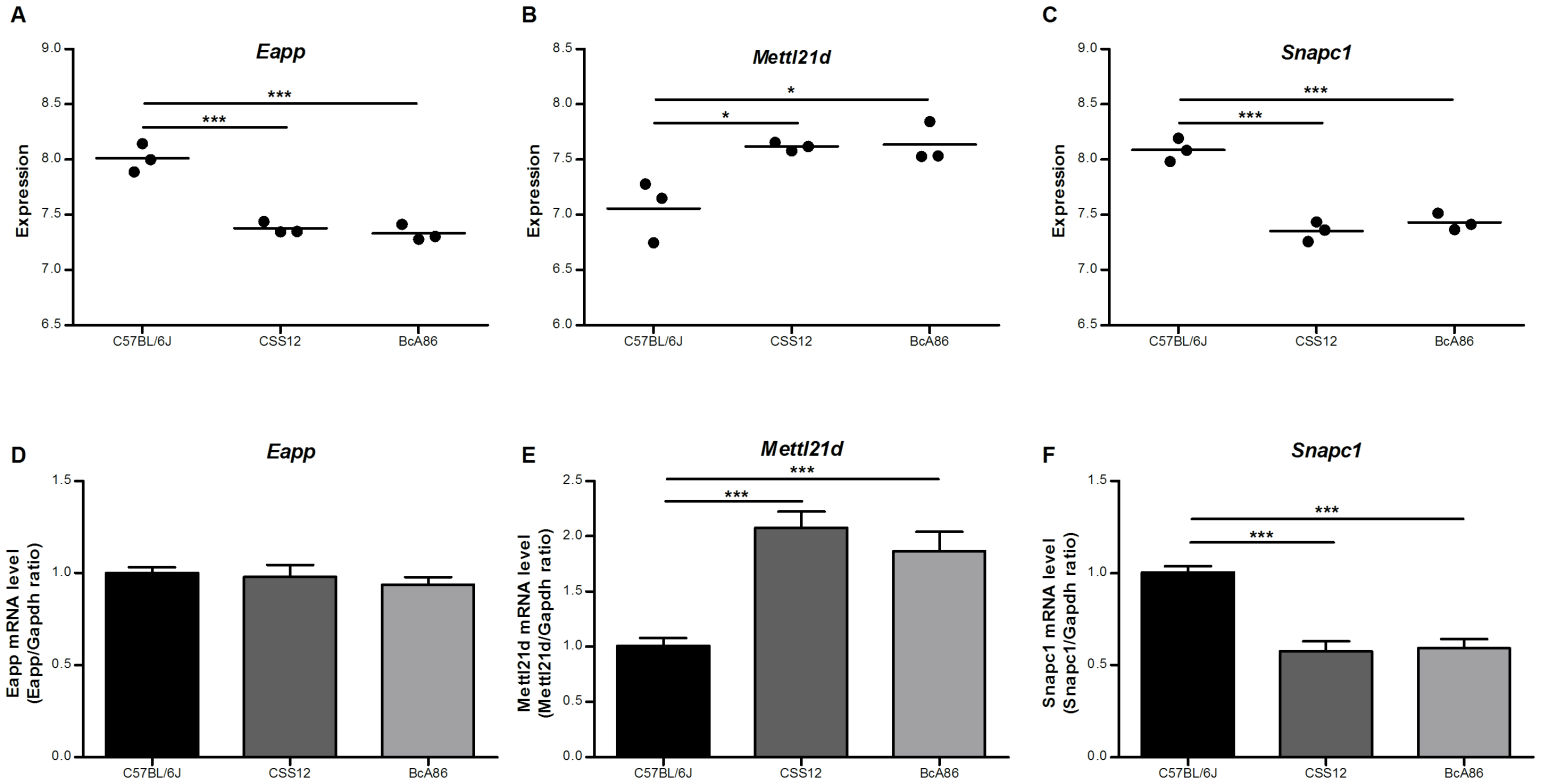
Selection of candidate genes within quantitative trait locus with expression differences. (A, B, and C) Dot plots of candidate genes from microarray analysis. Expression of *Eapp* and *Snapc1* was higher in hyporesponsive C57BL/6J strain than both hyperresponsive strains, CSS12 and BcA86. Conversely, the expression of *Mettl21d* was higher in CSS12 and BcA86 compared to C57BL/6J. (D, E, and F) Quantitative RT-PCR validates the microarray expression differences of *Mettl21* and *Snapc1*, but not of *Eapp*. Data are presented as mean ± SEM and statistical significance was calculated by one-way ANOVA and Bonferroni correction. n = 3 per strain for microarray and n>5 per strain for PCR; _*_ and _***_ represents *p*<0.05 and *p*<0.001 respectively.

From the microarray, *Eapp*, *Mettl21d*, and *Snapc1* were differentially expressed between the hyporesponsive strains and hyperresponsive strains, and also annotated as “validated” in the Reference Sequence database (Figure 5A-C). The differences observed in *Mettl21d* and *Snapc1*, but not *Eapp*, were confirmed by quantitative RT-PCR (Figure 5D-F). *Mettl21d* was expressed at higher levels in hyperresponsive strains, while the opposite was observed for *Snapc1*.

The QTL region associated with AHR was also mined for candidate genes that contain sequences variants that were predicted to have functional effect at the protein level. Within our QTL we identified 50 coding nsSNPs in 27 different genes that are possible candidates for causing variation in the AHR trait (Table 4). To reduce the number of candidates we used amino acid substitution prediction tools, PROVEAN and PolyPhen2, to determine which coding nsSNPs may affect the protein function from those that are neutral. Each tool uses its own algorithm based on protein sequence and/or structure to determine the effect. PROVEAN compares the mutated sequence to the reference sequence to calculate a delta alignment score. The score predicts the effect of an amino acid substitution in the context of its flanking sequences. A score of −2.5 was determined by PROVEAN creators as a default cutoff. Substitutions with scores less than −2.5 are considered deleterious, while substitutions with scores less than −4.1 are of greater confidence [19]. PolyPhen2 describes allele function as “benign”, “possibly damaging”, or the most confident, “probably damaging”. If a prediction cannot be made due to lack of sequence alignment data, then it is reported as “unknown”. Polyphen2 is based on comparison of sequence homology, three dimensional structure, and SWISS-PROT annotation of protein domains [20]. Genes with nsSNPs whose predicted effects are in consensus between both tools were selected as candidates.

**Table 4:** Sequence changes and PROVEAN scores of coding non-synonymous SNPs found in quantitative trait loci.

SNP ID	Gene	Entrez ID	PROVEAN	Polyphen2
rs29183105	Eapp	NP_079732.1	Neutral (−2.1)	**Probably damaging**
rs255321601	2700097O09Rik	NP_082590.2	Neutral (−0.38)	benign
rs46300008	Mipol1	NP_001157842.1	Neutral (1.625)	Benign
rs13481473	Mipol1	NP_001157842.1	Neutral (2.188)	Benign
rs13481474	Foxa1	NP_032285.2	**Deleterious (−4.76)**	**Probably damaging**
rs50478178	4921506M07Rik	NP_001032832.1	Neutral (2.842)	Benign
rs29124563	Ttc6	XP_003084948.1	**Deleterious (-3.192)**	Possibly damaging
rs31966428	Clec14a	NP_080085.3	Neutral (0.358)	Benign
rs13465063	Clec14a	NP_080085.3	Neutral (0.45)	Benign
rs13481500	Fscb	NP_001156743.1	Neutral (−0.62)	Unknown
rs29131205	Fscb	NP_001156743.1	Neutral (0.008)	Unknown
rs29133794	Fscb	NP_001156743.1	Neutral (−2.367)	Unknown
rs29192255	Fscb	NP_001156743.1	Neutral (1.763)	Unknown
rs29220106	Fscb	NP_001156743.1	Neutral (−1.764)	**Probably damaging**
rs33846378	Klhl28	NP_079983.1	Neural (−0.809)	Benign
rs212043559	Fancm	NP_849243.2	Neutral (−2.351)	**Probably damaging**
rs29212900	Fancm	NP_849243.2	Neutral (−0.478)	Possibly damaging
rs29213465	Fancm	NP_849243.2	Neutral (1.998)	Benign
rs29184120	Fancm	NP_849243.2	Neutral (−0.1)	Unknown
rs50634267	Mis18bp1	NP_766166.2	Neutral (0.757)	Benign
rs29135637	Pole2	NP_035263.1	Neutral (−0.169)	Benign
rs29193315	Atp5s	NP_080812.1	Neutral (0.505)	Benign
rs32225358	Nin	NP_032723.2	Neutral (−1.238)	Possibly damaging
rs29192398	Nin	NP_032723.2	Neutral (−1.57)	**Probably damaging**
rs29159683	Nin	NP_032723.2	Neutral (−1.851)	**Probably damaging**
rs29149025	Nin	NP_032723.2	Neutral (2.008)	Benign
rs29173916	Abhd12b	NP_001181962.1	Neutral (0.575)	Benign
rs13467444	Pygl	NP_573461.2	Neutral (−0.149)	Benign
rs47075219	Talpid3	NP_001156850.1	**Deleterious (-2.844)**	Unknown
rs29185339	Dact1	NP_067507.2	**Deleterious (-4.625)**	Benign
rs29222974	Dact1	NP_067507.2	Neutral (2.088)	Benign
rs3695552	Rtn1	NP_703187.2	Neutral (−1.15)	Possibly damaging
rs29198846	Lrrc9	NP_001136201.1	Neutral (−0.222)	Benign
rs29130757	Trmt5	NP_083856.1	Neutral (−2.496)	Benign
rs29166240	Trmt5	NP_083856.1	Neutral (−0.218)	**Probably damaging**
rs29162033	Trmt5	NP_083856.1	Neutral (−0.264)	Possibly damaging
rs29141846	Trmt5	NP_083856.1	Neutral (0.359)	Benign
rs13481528	Slc38a6	NP_001032806.2	Neutral (3.519)	Benign
rs50215140	Gm5068	XP_204772.4	Neutral (0.341)	Benign
rs236684046	Gm5068	XP_204772.4	Neutral (0.041)	Benign
rs48669127	Gm5068	XP_204772.4	Neutral (−0.052)	Possibly damaging
rs48452640	Gm5068	XP_204772.4	Neutral (−0.871)	Benign
rs32353362	Gm5068	XP_204772.4	Neutral (1.649)	Benign
rs32354318	Gm5068	XP_204772.4	Neutral (−1.92)	Benign
rs48608857	Gm5068	XP_204772.4	**Deleterious (-5.182)**	Possibly damaging
rs47247384	Gm5068	XP_204772.4	Neutral (−1.539)	Benign
rs29176285	Snapc1	NP_848479.1	Neutral (−0.926)	Benign
rs217102494	Dbpht2	NP_942566.1	Neutral (−0.5)	Unknown
rs29157778	Gpx2	NP_109602.2	Neutral (−0.296)	Benign
rs32685077	Smoc1	NP_071711.2	Neutral (0.365)	Benign

PROVEAN scores are in parentheses. SNPs are predicted to have functional effect by PROVEAN if they have a PROVEAN score of less than −2.5. SNPs. SNPs predicted to have functional effect are annotated as “deleterious” by PROVEAN and “probably damaging” by PolyPhen2 (bolded).

A number of nsSNPs were predicted as having a functional effect by either prediction tool (Table 4). PROVEAN classified the coding nsSNPs in *Foxa1*, *Ttc6*, *Talpid3*, *Dact1*, and *Gm5068* as “deleterious”. PolyPhen2 classified the coding nsSNPs in *Eapp*, *Foxa1*, *Fscb*, *Fancm*, *Nin* and *Trmt5* as “probably damaging”. The coding nsSNP in *Foxa1* was the only one predicted to have functional effect by both tools. Taken together, based on analysis of expression differences and of nsSNPs we select *Foxa1*, *Mettl21d*, and *Snapc1* as candidate genes for airway responsiveness.

## Discussion

There have been many attempts to map QTLs for AHR using murine crosses resulting in associations with loci on Chromosomes 2, 6, 11, 15, 17, and 18 [3]–[5]. In our previous study, phenotype-genotype association analysis of all 33 strains in our AcB/BcA panel identified 16 chromosomal segments that were significantly associated with airway responsiveness. The 33 strains in the AcB/BcA panel are all informative since their recombinant regions cause their airway responsiveness phenotypes to vary from the phenotypes of their respective major genetic donor strains. Therefore, each strain can be further explored by producing F2 progeny to identify the genetic loci associated with their phenotypes.

### A/J Chromosome 12 is associated with airway hyperresponsiveness

We selected the BcA86 strain for F2 mapping because it had the most significantly different phenotype compared to C57BL/6J, its major genetic donor strain. We believe that our results here represent a significant advancement from our last study in which we identified 16 regions associated with AHR.

The BcA86 genome contains eight recombinant A/J regions, and five of them overlap with the 16 regions we previously associated with AHR [15]. In this study we were able to statistically determine that the BcA86 recombinant region on Chromosome 12 showed strongest association with AHR. The recombinant A/J region on Chromosome 12 of the BcA86 genome was previously genotyped to be from 33.38 Mbp to the end of the chromosome [10]. Through phenotype-genotype statistical analysis of an F2 population of BcA86 we determined that a 28Mbp QTL, spanning from 54.6 to 82.6 Mbp is associated with airway responsiveness. Specifically, the A/J allele at this QTL contributes to higher airway responsiveness. In addition, the QTL for AHR identified in this study validates one of the 16 chromosomal segments we previously associated with AHR when studying the entire RCS panel as a whole [15]. This region explains 12.5% of the variance in the AHR phenotype in the BcA86F2 mice. Although we only identified one QTL on Chromosome 12, we cannot reject the hypothesis that other loci could be interacting with this region. Other loci, either independently or interacting with the QTL on Chromosome 12, could explain the rest of the variance. Our model did not have enough power to detect interacting loci through multiple-QTL analysis.

Our results from experiments with consomic CSS12 confirm the importance of mouse Chromosome 12 in determining the airway responsiveness phenotype. BcA86 has recombinant regions in many chromosomes, but only Chromosome 12 contained markers that were significantly associated with AHR after QTL analysis. CSS12 mice have a phenotype similar to mice from the hyperresponsive A/J and BcA86 strains, proving that Chromosome 12 of A/J origin is alone sufficient to create the AHR phenotype in a C57BL/6J background. The opposite, whether mice with C57BL/6J Chromosome 12 in the context of an A/J background would have lower airway responsiveness phenotype than A/J mice, still remains to be explored. Furthermore, the regions identified to be linked to AHR using BcA86F2 mice may be further validated and the size can be reduced through QTL analysis of F2 mice generated from CSS12 and C57BL/6J.

In order to observe the AHR phenotype in CSS12 mice by measuring Penh, we performed a methacholine dose response curve. A 6.25 mg/ml dose of methacholine was enough to observe the difference in phenotypes between BcA86 and C57BL/6J strains. However, to observe a difference between C57BL/6J and CSS12 strains, higher doses of methacholine (25 and 50 mg/ml) were needed as the differences in their phenotypes were more subtle. There are several possible explanations for this difference in phenotype between BcA86 and CSS12 strains. CSS12 has only one donor strain region on a C57BL/6J background, while BcA86 has several recombinant regions. Interactions between the Chromosome 12 QTLs and other recombinant regions might be causing the phenotype to be more readily observed in BcA86. The presence of multiple interacting recombinant regions in BcA86 also explains the transgressive pattern of segregation in the phenotype of the BcA86F2 mice, as some mice had higher and lower values of Penh than either parental strain. In addition, the BcA86 strain has been inbred for many generations and new mutations, not present in A/J or CSS12, might be found in the BcA86 genome and contribute to its stronger phenotype.

### Airway hyporesponsive and hyperresponsive strains can be distinguished by pathway analysis

We did a pathway analysis to understand why CSS12 and BcA86 have such a different airway responsiveness phenotype compared to their major genetic background strain. Genes differentially expressed between the hyporesponsive and hyperresponsive strains categorize into networks related to “cell mediated immune responses” encompassing functions such as survival and proliferation of blood cells, and development of muscle and connective tissue cells. Given that AHR is mediated by the contraction of smooth muscle cells and the elastic component of the connective tissue surrounding the airways, we believe that functions involved in proper muscle development are good candidate pathways to explain the phenotypic distinction. Inflammatory cells found in the blood can also localize in the airway connective tissue and contribute to the phenotype. Our list of genes distinguishing hyperresponsive from hyporesponsive strains was significantly associated with the canonical pathway of “airway pathology in COPD”. Like asthma, COPD is defined by inflammation and remodeling of the airways and AHR is a phenotype observed in COPD patients as well. Genes and networks common to both lung diseases have been identified [22]. *Mmp2* is the primary gene associating our list of 105 genes to this canonical pathway, and *Mmp2* expression, which is elevated in our hyperresponsive strains, has been positively correlated with allergen induced airway remodeling that can lead to AHR [23].

### Potential candidate genes for airway responsiveness

Our initial approach aimed at gaining mechanistic insight into why BcA86 and CSS12 are different from C57BL/6J was to perform microarray analyses. Using this method, we identified two candidate genes which were within our QTL, *Mettl21d* and *Snapc1,* whose expressions were different between hyporesponsive and hyperresponsive strains of mice. However, since more than 50% of mutations involved in inherited diseases are coding nsSNPs, our second approach was to select genes that have coding nsSNPs between the A/J and C57BL/6J sequences that are predicted to affect protein function. Within our AHR associated loci, there are 50 coding nsSNPs in 27 different genes. We used two different tools to predict the effects of the nsSNPs because each tool uses unique algorithms to make its predictions. Amino acid substitution prediction tools can be based on information gathered on protein sequence, domains and structure. Each tool has its own strengths and weakness; therefore, we combined the results obtained from the two different tools to formulate our list of candidate genes. Briefly, the foundation of the PROVEAN algorithm is based on sequence homology and the notion that proteins belonging to a family have amino acid sequences that are evolutionarily conserved between members and across species [19]. The PolyPhen2 algorithm is based on sequence conservation and protein structure annotation [20]. We considered nsSNPs with predictions that were in consensus between both tools to be of greater confidence to have a functional effect. Based on the results of our analysis *Foxa1* was our best candidate gene containing a coding nsSNP. Overall, our final list of candidate genes for AHR selected based on expression or sequence differences contains *Mettl21d Snapc1*, and *Foxa1*.

*Mettl21d* is a lysine methyltransferase and its methylation activity has only been shown on ATP catalyzing chaperone, *VCP*, to reduce its activity at its ATPase domain 1 [24]. *VCP* participates and provides energy for the endoplasmic reticulum associated degradation pathway to rid the cell of misfolded proteins and prevent cellular stress.

*Snapc1* is a 43kDa protein that is part of the small nuclear activating protein complex (SNAPc) and associates with RNA polymerase II and III to initiate transcription of genes. The promoters of 267 genes were identified as bound by SNAPC1 and RNA polymerase II [25]. A handful of SNAPC1 targets have already been associated with airway responsiveness and allergic asthma phenotypes. An example is *NFΚBIA* which has been associated with susceptibility to atopic asthma [26], childhood asthma, AHR, and bronchopulmonary dysplasia in pediatric lung disease cohorts [27].

*Foxa1* belongs to the forkhead box (FOX) class of transcription factors, which includes at least 40 members united by an evolutionarily conserved DNA binding domain of about 100-amino acids in length, known as the forkhead domain [28]. The prediction that rs13481474 will have an effect on the function of FOXA1 remains to be validated. This mutation lies within the well characterized C-terminal region of the protein [29]. Deletion of the C-terminal region has been shown to affect the transcriptional activity of the protein, which binds to the promoters of more than 100 genes involved in various processes, including metabolism, development, cell cycle, and enzyme activity [30]–[33].

Foxa1 has been shown to be expressed in epithelial cells lining the airways of the mouse and human fetus since lung bud formation, and lack of the gene stops proliferation and differentiation of lung cells and airway branching [34], [35]. Human studies have also pointed to *FOXA1* as a susceptibility locus. A meta-analysis study involving subjects with allergy and asthma identified the Chromosome 14q21.1 region and *FOXA1* as candidates [36]. Through another genome wide study, *FOXA1* was associated with lung function decline in COPD based on its proximity to the risk loci, and FOXA1 expression was different between the lungs of cases with and without airway obstruction [37].

## Conclusion

Overall, the present study demonstrates how changing the genotype of Chromosome 12 in C57BL/6J mice can affect the airway responsiveness phenotype. By QTL mapping in F2 mice from a RCS we deduced that the regions from 54.6 to 82.6 Mbp on Chromosome 12 is statistically associated with AHR. Genes within these regions can be mined via many strategies for candidate genes involved in AHR. We identified *Mettl21d*, *Snapc1* and *Foxa1* as candidates for airway responsiveness based on expression and sequence differences between the complementary strains we used to study airway responsiveness. Using similar approaches used to study BcA86, the other informative strains in the RCS panel can be studied to build a network of candidate regions and genes associated with the airway responsiveness complex trait.

## Supporting Information

Table S1 **Markers genotyped in BcA86F2 mice and their LOD scores.**
(XLSX)

Table S2 **Differently expressed genes between hyporesponsive strain (C57BL/6J) and hyperresponsive strains (BcA86 and CSS12).**
(XLSX)

## References

[1] Levitt RC, Mitzner W (1989) Autosomal recessive inheritance of airway hyperreactivity to 5-hydroxytryptamine. J Appl Physiol 67: 1125–1132. 2793705 10.1152/jappl.1989.67.3.1125

[2] Leme AS, Berndt A, Williams LK, Tsaih SW, Szatkiewicz JP, et al (2010) A survey of airway responsiveness in 36 inbred mouse strains facilitates gene mapping studies and identification of quantitative trait loci. Mol Genet Genomics 283: 317–326. 20143096 10.1007/s00438-010-0515-x PMC2885868

[3] De Sanctis GT, Merchant M, Beier DR, Dredge RD, Grobholz JK, et al (1995) Quantitative locus analysis of airway hyperresponsiveness in A/J and C57BL/6J mice. Nat Genet 11: 150–154. 7550342 10.1038/ng1095-150

[4] Ackerman KG, Huang H, Grasemann H, Puma C, Singer JB, et al (2005) Interacting genetic loci cause airway hyperresponsiveness. Physiol Genomics 21: 105–111. 15657107 10.1152/physiolgenomics.00267.2004

[5] Ferreira CM, Chen JL, Li J, Shimomura K, Yang X, et al (2012) Genetic interactions between chromosomes 11 and 18 contribute to airway hyperresponsiveness in mice. PLoS One 7: e29579. 22253740 10.1371/journal.pone.0029579 PMC3254621

[6] De Sanctis GT, Singer JB, Jiao A, Yandava CN, Lee YH, et al (1999) Quantitative trait locus mapping of airway responsiveness to chromosomes 6 and 7 in inbred mice. Am J Physiol 277: L1118–L1123. 10600881 10.1152/ajplung.1999.277.6.L1118

[7] Ewart SL, Mitzner W, DiSilvestre DA, Meyers DA, Levitt RC (1996) Airway hyperresponsiveness to acetylcholine: segregation analysis and evidence for linkage to murine chromosome 6. Am J Respir Cell Mol Biol 14: 487–495. 8624254 10.1165/ajrcmb.14.5.8624254

[8] Nicolaides NC, Holroyd KJ, Ewart SL, Eleff SM, Kiser MB, et al (1997) Interleukin 9: a candidate gene for asthma. Proc Natl Acad Sci U S A 94: 13175–13180. 9371819 10.1073/pnas.94.24.13175 PMC24282

[9] Fortin A, Diez E, Rochefort D, Laroche L, Malo D, et al (2001) Recombinant congenic strains derived from A/J and C57BL/6J: a tool for genetic dissection of complex traits. Genomics 74: 21–35. 11374899 10.1006/geno.2001.6528

[10] Boivin GA, Pothlichet J, Skamene E, Brown EG, Loredo-Osti JC, et al (2012) Mapping of clinical and expression quantitative trait loci in a sex-dependent effect of host susceptibility to mouse-adapted influenza H3N2/HK/1/68. J Immunol 188: 3949–3960. 22427645 10.4049/jimmunol.1103320

[11] Fortin A, Cardon LR, Tam M, Skamene E, Stevenson MM, et al (2001) Identification of a new malaria susceptibility locus (Char4) in recombinant congenic strains of mice. Proc Natl Acad Sci U S A 98: 10793–10798. 11535821 10.1073/pnas.191288998 PMC58554

[12] Roy MF, Riendeau N, Loredo-Osti JC, Malo D (2006) Complexity in the host response to Salmonella Typhimurium infection in AcB and BcA recombinant congenic strains. Genes Immun 7: 655–666. 17024130 10.1038/sj.gene.6364344

[13] Gill K, Boyle AE (2005) Genetic analysis of alcohol intake in recombinant inbred and congenic strains derived from A/J and C57BL/6J progenitors. Mamm Genome 16: 319–331. 16104380 10.1007/s00335-004-2239-x

[14] Gill KJ, Boyle AE (2005) Quantitative trait loci for novelty/stress-induced locomotor activation in recombinant inbred (RI) and recombinant congenic (RC) strains of mice. Behav Brain Res 161: 113–124. 15904718 10.1016/j.bbr.2005.01.013

[15] Camateros P, Marino R, Fortin A, Martin JG, Skamene E, et al (2010) Identification of novel chromosomal regions associated with airway hyperresponsiveness in recombinant congenic strains of mice. Mamm Genome 21: 28–38. 20012967 10.1007/s00335-009-9236-z

[16] Irizarry RA, Hobbs B, Collin F, Beazer-Barclay YD, Antonellis KJ, et al (2003) Exploration, normalization, and summaries of high density oligonucleotide array probe level data. Biostatistics 4: 249–264. 12925520 10.1093/biostatistics/4.2.249

[17] Blazejczyk M, Miron M, Nadon R (2007) FlexArray: A Statistical Data Analysis Software for Gene Expression Microarrays., version Génome Québec, Montreal, Canada.

[18] Pruitt KD, Tatusova T, Maglott DR (2007) NCBI reference sequences (RefSeq): a curated non-redundant sequence database of genomes, transcripts and proteins. Nucleic Acids Res 35: D61–D65. 17130148 10.1093/nar/gkl842 PMC1716718

[19] Choi Y, Sims GE, Murphy S, Miller JR, Chan AP (2012) Predicting the functional effect of amino acid substitutions and indels. PLoS One 7: e46688. 23056405 10.1371/journal.pone.0046688 PMC3466303

[20] Adzhubei IA, Schmidt S, Peshkin L, Ramensky VE, Gerasimova A, et al (2010) A method and server for predicting damaging missense mutations. Nat Methods 7: 248–249. 20354512 10.1038/nmeth0410-248 PMC2855889

[21] Bates J, Irvin C, Brusasco V, Drazen J, Fredberg J, et al (2004) The use and misuse of Penh in animal models of lung disease. Am J Respir Cell Mol Biol 31: 373–374. 15317683 10.1165/ajrcmb.31.3.1

[22] Kaneko Y, Yatagai Y, Yamada H, Iijima H, Masuko H, et al (2013) The search for common pathways underlying asthma and COPD. Int J Chron Obstruct Pulmon Dis 8: 65–78 doi: 10.2147/COPD.S39617. Epub@2013 Jan 25.: 65–78 23378757 10.2147/COPD.S39617 PMC3558318

[23] Firszt R, Francisco D, Church TD, Thomas JM, Ingram JL, et al.. (2013) Interleukin-13 induces collagen type-1 expression through matrix metalloproteinase-2 and transforming growth factor-beta1 in airway fibroblasts in asthma. Eur Respir J. 10.1183/09031936.00068712 PMC6747688 23682108

[24] Kernstock S, Davydova E, Jakobsson M, Moen A, Pettersen S, et al (2012) Lysine methylation of VCP by a member of a novel human protein methyltransferase family. Nat Commun 3: 1038 doi: 10.1038/ncomms2041.: 1038 22948820 10.1038/ncomms2041

[25] Baillat D, Gardini A, Cesaroni M, Shiekhattar R (2012) Requirement for SNAPC1 in transcriptional responsiveness to diverse extracellular signals. Mol Cell Biol 32: 4642–4650. 22966203 10.1128/MCB.00906-12 PMC3486178

[26] Daley D, Park JE, He JQ, Yan J, Akhabir L, et al (2012) Associations and interactions of genetic polymorphisms in innate immunity genes with early viral infections and susceptibility to asthma and asthma-related phenotypes. J Allergy Clin Immunol 130: 1284–1293. 23063165 10.1016/j.jaci.2012.07.051

[27] Ali S, Hirschfeld AF, Mayer ML, Fortuno ES III, Corbett N, et al (2013) Functional genetic variation in NFKBIA and susceptibility to childhood asthma, bronchiolitis, and bronchopulmonary dysplasia. J Immunol 190: 3949–3958. 23487427 10.4049/jimmunol.1201015

[28] Jackson BC, Carpenter C, Nebert DW, Vasiliou V (2010) Update of human and mouse forkhead box (FOX) gene families. Hum Genomics 4: 345–352. 20650821 10.1186/1479-7364-4-5-345 PMC3500164

[29] Pani L, Overdier DG, Porcella A, Qian X, Lai E, et al (1992) Hepatocyte nuclear factor 3 beta contains two transcriptional activation domains, one of which is novel and conserved with the Drosophila fork head protein. Mol Cell Biol 12: 3723–3732. 1324404 10.1128/mcb.12.9.3723-3732.1992 PMC360231

[30] Wolf I, Bose S, Williamson EA, Miller CW, Karlan BY, et al (2007) FOXA1: Growth inhibitor and a favorable prognostic factor in human breast cancer. Int J Cancer 120: 1013–1022. 17163418 10.1002/ijc.22389

[31] Gao N, Zhang J, Rao MA, Case TC, Mirosevich J, et al (2003) The role of hepatocyte nuclear factor-3 alpha (Forkhead Box A1) and androgen receptor in transcriptional regulation of prostatic genes. Mol Endocrinol 17: 1484–1507. 12750453 10.1210/me.2003-0020

[32] Carlsson P, Mahlapuu M (2002) Forkhead transcription factors: key players in development and metabolism. Dev Biol 250: 1–23. 12297093 10.1006/dbio.2002.0780

[33] Tomaru Y, Kondo S, Suzuki M, Hayashizaki Y (2003) A comprehensive search for HNF-3alpha-regulated genes in mouse hepatoma cells by 60K cDNA microarray and chromatin immunoprecipitation/PCR analysis. Biochem Biophys Res Commun 310: 667–674. 14521963 10.1016/j.bbrc.2003.08.148

[34] Wan H, Dingle S, Xu Y, Besnard V, Kaestner KH, et al (2005) Compensatory roles of Foxa1 and Foxa2 during lung morphogenesis. J Biol Chem 280: 13809–13816. 15668254 10.1074/jbc.M414122200

[35] Besnard V, Wert SE, Hull WM, Whitsett JA (2004) Immunohistochemical localization of Foxa1 and Foxa2 in mouse embryos and adult tissues. Gene Expr Patterns 5: 193–208. 15567715 10.1016/j.modgep.2004.08.006

[36] Hinds DA, McMahon G, Kiefer AK, Do CB, Eriksson N, et al (2013) A genome-wide association meta-analysis of self-reported allergy identifies shared and allergy-specific susceptibility loci. Nat Genet 45: 907–911. 23817569 10.1038/ng.2686 PMC3753407

[37] Hansel NN, Ruczinski I, Rafaels N, Sin DD, Daley D, et al (2013) Genome-wide study identifies two loci associated with lung function decline in mild to moderate COPD. Hum Genet 132: 79–90. 22986903 10.1007/s00439-012-1219-6 PMC3536920

